# Canine Adenovirus 2: A Natural Choice for Brain Circuit Dissection

**DOI:** 10.3389/fnmol.2020.00009

**Published:** 2020-02-27

**Authors:** Andréanne Lavoie, Bao-hua Liu

**Affiliations:** ^1^Department of Biology, University of Toronto Mississauga, Mississauga, ON, Canada; ^2^Department of Cell and Systems Biology, University of Toronto, Toronto, ON, Canada

**Keywords:** canine adenovirus 2, neural circuit, retrograde, circuit tracing, circuit function, cell type specificity, projection specificity

## Abstract

Canine adenovirus-2 (CAV) is a canine pathogen that has been used in a variety of applications, from vaccines against more infectious strains of CAV to treatments for neurological disorders. With recent engineering, CAV has become a natural choice for neuroscientists dissecting the connectivity and function of brain circuits. Specifically, as a reliable genetic vector with minimal immunogenic and cytotoxic reactivity, CAV has been used for the retrograde transduction of various types of projection neurons. Consequently, CAV is particularly useful when studying the anatomy and functions of long-range projections. Moreover, combining CAV with conditional expression and transsynaptic tracing results in the ability to study circuits with cell- and/or projection-type specificity. Lastly, with the well-documented knowledge of viral transduction, new innovations have been developed to increase the transduction efficiency of CAV and circumvent its tropism, expanding the potential of CAV for circuit analysis.

## Introduction

Canine adenovirus-2 (CAV) is a non-human pathogen that causes a mild infectious respiratory disease in dogs known as “kennel cough.” CAV was initially studied to develop a vaccine against canine adenoviruses-1, the cause of a severe liver disease in dogs, and proposed as a long-term gene therapy vector for neurological disorders (Kremer et al., 2000; Chillon and Kremer, 2001; Soudais et al., 2001). Since then, CAV has also attracted neuroscientists because of several advantageous properties for studying the anatomy and function of neural circuits (Junyent and Kremer, 2015; del Rio et al., 2019). Specifically, it is primarily the retrograde ability of CAV to deliver cargo genes via axon terminals that allow the investigation of the pattern and function of long-range projections (Junyent and Kremer, 2015). In addition, CAV is useful for studying neural circuits because it has a high neuronal specificity and a strong compatibility with conditional gene expression (Kremer et al., 2000; Soudais et al., 2001; Junyent and Kremer, 2015). Furthermore, CAV is a reliable vector for long-term functional studies, since it mediates stable, lasting gene expression while sustaining low immunogenicity and cytotoxicity levels (Kremer et al., 2000; Chillon and Kremer, 2001; Soudais et al., 2001). This mini-review will summarize the properties that give CAV its edge, highlighting their utility in dissecting the connectivity and functions of neural circuits.

## Beneficial Properties of CAV for Circuit Analysis

Canine adenovirus-2 viruses have been widely utilized in neuroscience due to their capability of infecting axon terminals via a retrograde mechanism and then driving gene expression at the somata. Owing to this retrograde capability, when injected into a brain region, CAV viruses transduce projection neurons which innervate the injection site, in addition to neurons at the injection site (Kremer et al., 2000; Chillon and Kremer, 2001; Soudais et al., 2001; Bru et al., 2010). CAV’s retrograde capability relies on the coxsackievirus and adenovirus receptor (CAR) (Kremer et al., 2000; Chillon and Kremer, 2001; Soudais et al., 2001). CAR is a cell adhesion molecule necessary for the docking, internalization, endocytosis, and axonal transport of CAV viruses (Salinas et al., 2009). This receptor is highly enriched at presynaptic sites of neurons, but exists in low density in the somata and dendrites of neurons (Zussy et al., 2016). This domain-specific CAR expression gives rise to the retrograde transport of CAV, making it a powerful tool for mapping long-range connectivity between brain regions (Junyent and Kremer, 2015).

Another advantage of CAV for neuroscientists is that CAV vectors exhibit strong tropism, biasing neurons. For example, in the peripheral nervous system, CAV viruses preferentially transduced olfactory sensory neurons instead of the columnar epithelial cells (Bru et al., 2010). Moreover, when injected in the central nervous system, CAV strongly infects neurons but not non-neuronal types (Soudais et al., 2001; Bru et al., 2010). The molecular basis for this neuronal tropism is that CAR expression in the brain is primarily, if not exclusively, on neurons but not on astrocytes, oligodendrocytes, endothelium, or meningeal cells (Soudais et al., 2001; Persson et al., 2006). So far, in the central nervous system, CAV vectors have been successfully used to transduce various types of neurons in broad regions of the brain, demonstrating their wide applicability for studying neural circuits (Table 1). For example, CAV can transduce a variety of neurotransmitter systems, including, but not limited to, glutamatergic, dopaminergic, GABAergic, noradrenergic, oxytonergic, serotonergic, and cholinergic systems (Table 1). Furthermore, CAV has been successfully used in a broad diversity of cortical and subcortical projection pathways (Table 1). Even though rodents were used as the animal models in most of CAV applications in Table 1, recently, CAV was also successfully used in non-human primates (Table 1; Mestre-Francés et al., 2018; Bohlen et al., 2019; Dopeso-Reyes et al., 2019). These studies validate the applicability of CAV as a gene delivery tool in non-human primates, facilitating the investigation of neural circuits in a more human-relevant model.

**TABLE 1:** Summary of cell types and circuit pathways where CAV had been used for circuit analysis.

System	Circuit	Effector	References
Cholinergic	DBB → arcuate nucleus	tdTomato	Herman et al., 2016
	LDTg → VTA	hM3D(Gq)-mCherry	Fernandez et al., 2018
	nuclei of Meynert → striatum	GFP	Soudais et al., 2004
Dopaminergic	SNc → striatum	GFP	Soudais et al., 2004
		TH	Sotak et al., 2005
			Robinson et al., 2007
		RG, TVA-mCherry, GFP, tdTomato, mGFP, SYP-mRuby, GCaMP6f	Lerner et al., 2015
	VTA → NAc	ArchT-GFP	Luo et al., 2018
		hM3D(Gq)-mCherry,	Boender et al., 2014
		hM3D(Gq)-mCherry,	Kakava-Georgiadou et al., 2019
		GFP, NBL10	Ekstrand et al., 2014
		eYFP	Reynolds et al., 2018
	VTA → (NAc, mPFC, Amy)	GFP	Beier et al., 2015
	(VTA, SNc) → Striatum	TH	Hnasko et al., 2006
	VTA → (NAc, BLA)		Fadok et al., 2010
	Midbrain human organoid	GFP	Brito et al., 2012
			Simão et al., 2016;
GABAergic	POA → (TMN, PFC)	GFP, RG,TVA-mCherry, mGFP, SYP-mRuby	Chung et al., 2017
	mPOA → (PAG, PVN, Amy, VTA)	ZsGreen, mCherry, myrGFP, RG, TVA-mCherry	Kohl et al., 2018
	CeA → (PCRt, PAG)	ChR2-mCherry, GFP	Han et al., 2017
	CeA→ PAG	RG, ArchT-GFP	Xu et al., 2016
	Cervical → lumbar	tdTomato, DTR, SynTag	Ruder et al., 2016
Glutamatergic	PLC → (NAc, Amy)	GCaMP6f, GFPL10	Murugan et al., 2017
	PLC → PVT	hM3D(Gq)-mCherry, hM4D(Gi)-mCherry	Campus et al., 2019
	GC → BLA	tdTomato	Lavi et al., 2018
	(HPC, mPFC) → LS	GFP, hM3D(Gq)-mCherry, hM4D(Gi)-mCherry	Parfitt et al., 2017
	Cervical → lumbar	tdTomato, DTR, SynTag	Ruder et al., 2016
	mPFC → PAG	ChR2-YFP	Vander Weele et al., 2018
	mPFC → NAc	hM3D(Gq)-mCherry, ChR2-eYFP	Augur et al., 2016
	mPFC → RE	hM4D(Gi)-mCherry	Ramanathan et al., 2018
	mPFC → MDT; MDT→ mPFC	hM4D(Gi)-mCherry	Alcaraz et al., 2018
	(contra. mPFC, MDT, HPC) → mPFC	hM3D(Gq)-mCherry, tdTomato	Miller et al., 2017
	PFC → (NAc, PVT)	GCaMP6; eYFP, ChR2-eYFP, eNpHR-eYFP	Otis et al., 2017
	ACC → HPC	eNpHR, eYFP	Rajasethupathy et al., 2015
	(ORBvl, VC, RSP, contra. ACC) → ACC	tdTomato	Chatterjee et al., 2018
	OFC → striatum	ChR2-eYFP	Hirokawa et al., 2019
	VC → NOT/DTN	tdTomato, DTR-GFP	Liu et al., 2016
	VC → SC	tdTomato	Zingg et al., 2017
	ACtx → IC	GCaMP6s	Asokan et al., 2018
		ChR2-mCherrry	Williamson and Polley, 2019
	(ACtx, Ath) → striatum	tdTomato, ChR2	Ponvert and Jaramillo, 2019
	MC → (contra. MC, Me) (OB, HPC, ACtx, Me) → LC	RG, GFP, TVA-mCherry	Schwarz et al., 2015
	MC → (Spinal cord, Contra. MC)	GCaMP6s, tdTomato	Kim et al., 2017
	EC → DG	ChR2-YFP, tdTomato	Leroy et al., 2017
	Periform cortex → (OB, CoA)	ChR2-YFP	Diodato et al., 2016
	Multiple cortices → BPN	eGFP	Tervo et al., 2016
	PBNl → (CeA, MdD)	hM4D(Gi)-mCherry	Carter et al., 2013
		hM4D(Gi)-mCherry, mCherry	Alhadeff et al., 2017
		hM3D(Gq), PLAP	Barik et al., 2018
	vMT → (BLA, mPFC, NAc)	hM3D(Gq)-mCherry	Salay et al., 2018
	BLA → mPFC	tdTomato	Vogel et al., 2016
		ChR2-eNpHR-venus	Senn et al., 2014
		ChR2-mRubby, DsRed, GFP	Li et al., 2018
	BLA → (NAc, CeA, HPC)	ChR2-eYPF	Beyeler et al., 2016, 2018
		eNpHR-eYFP, eYFP	Namburi et al., 2015
	(Multiple cortices, SNc, basal nuclei of Meynert, Thalamic nuclei) → Striatum	GFP, LRRK2	Mestre-Francés et al., 2018*
	(DCN, IO, VN, NRTP, etc.) → Cb	GFP	Dopeso-Reyes et al., 2019*
	Pontine nuclei → vermis lobule	GFP, RG, TVA-mCherry	Wagner et al., 2019
	LH → LHb	hM4D(Gi)-mCherry, tdTomato	Lecca et al., 2017
	CLA → mPFC	tdTomato	Ährlund-Richter et al., 2019
	HPC → (CeA, BLA)	ChR2-eYFP, RG, TVA, ArchT-GFP, GFP, eNpHR-eYFP	Xu et al., 2016
	POm → M1	RG, GFP, TVA-mCherry	Mo and Sherman, 2019
	PVT → NAc	GCamP6s	Otis et al., 2019
	preBötC → LC	DTR	Yackle et al., 2017
Noradrenergic	LC → spinal cord	ChR2-mCherry	Li et al., 2016
	LC → (spinal cord, PFC)	PSAM-eGFP	Hirschberg et al., 2017
	LC → (ACtx, Cb, HPC, Me, OB)	RG, GFP, TVA-mCherry	Schwarz et al., 2015
	LC → (BLA, mPFC)	ChR2, mAG1, SYP-mCherry, ArchT-tdTomato	Uematsu et al., 2017
Oxytonergic	PVN → SON	eGFP; SYP-GFP ChR2-mCherry, hM4D(Gi)-mCherry	Eliava et al., 2016
Serotonergic	DRN → Amy	5-HT1b, GFP	Liu et al., 2015
	Raphe nuclei → NTS	Knockout Tph2	Wu et al., 2012
Non-canonical neurotransmitters	PVT → CeA	hM4D(Gi)-mCherry	Penzo et al., 2015
	CeA → LC	DTX	Andreoli et al., 2017
Periphery	Motor neurons → craniofacial muscle	GFP	Bohlen et al., 2019*
	NG → gut	GFP, rM3D(Gs)-mCherry	Han et al., 2018

The citations in this table serve as examples of each type of studies. *Non-human primate studies.

**FIGURE 1:**
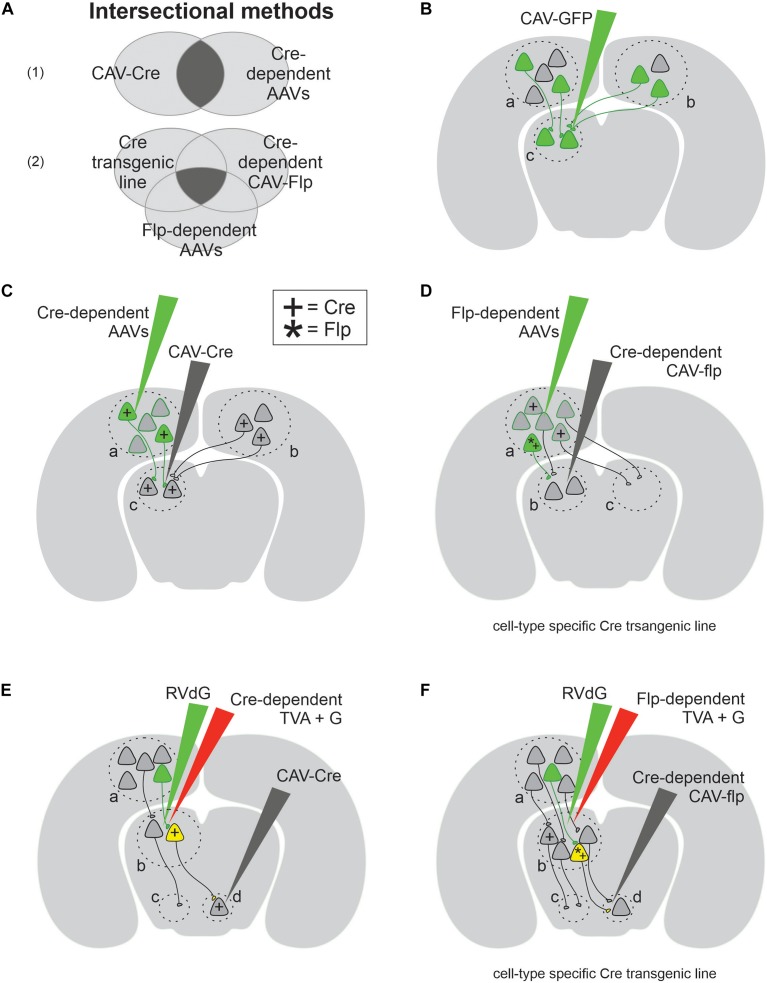
Canine adenovirus type 2 (CAV-2) combined with genetic approaches allows a variety of experimental designs for circuit analyses. **(A)** Venn diagram of two intersectional methods: part **(A1)** provides projection-specific expression using two conditions, while part **(A2)** provides cell-type and projection-specific expression using three conditions. **(B)** The simplest experimental design that labels both local (area c) and projection neurons (areas a and b). **(C)** The schematics of experimental designs for projection-specific expression with the help of Cre (+) recombinase. **(D)** The schematics of experimental designs for cell-type and projection-specific expression with the help of both Cre (+) and Flp (*) recombinases. **(E)** The schematics of transsynaptic experimental design which selectively labels the input onto projection-specific neuronal population defined by retrograde CAV-Cre. **(F)** The schematics of transsynaptic experimental design which selectively labels the input onto cell-type and projection-specific neuronal population.

Among a number of retrograde viruses (for example, rabies, lentivirus, and rAAV2-retro), what makes CAV viruses unique is their carrying capacity and physical size. In fact, CAV is the second largest retrograde virus with a 30–36 kb cloning capacity and ∼90 nm diameter, leading over rabies (1–3 kb) and lentivirus (9 kb) by 10- and 3-fold, respectively (Thompson and Towne, 2018). The large carrying load of CAV allows flexible, diverse, and creative design of cargo genes, which is particularly useful when dissecting neural circuits. For example, one can combine a variety of genes such as optogenetic tools, genetically encoded indicators of neuronal activity, fluorophores, large promoters, recombinases, LoxP/Flp sequences, and so on in one CAV vector (Figure 1A). Such combinations allow one to selectively label neurons according to cell type and/or projection, and they also facilitate efforts to monitor and perturb neural activity simultaneously (Soudais et al., 2004; Junyent and Kremer, 2015). Moreover, the large physical size of CAV provides advantages for neuroscientists performing stereotactic injections into small nuclei. CAV viruses (∼90 nm in diameter) remain near the injection site compared to smaller adeno-associated viruses (AAV) (22 nm in diameter). For instance, 0.25–0.5 μl of CAV spread on average by 200 μm from the center of the injection site, while 0.25 μl of rAAV2-retro spread four times more (Schwarz et al., 2015; Tervo et al., 2016). This restricted diffusion of CAV is useful when studying small structures, such as the complex of the nucleus of the optic tract and the dorsal terminal nucleus (NOT/DTN) (Liu et al., 2016), PAG (de Git et al., 2018) or CLA (Crick and Koch, 2005). It is worth noting that CAV cannot be injected with iontophoresis, which is commonly used to confine the spread of electrically charged AAV viruses (Gerhardt and Palmer, 1987), because the coat proteins of CAV are almost electrically neutral (10 times less charged than AAV viruses) (Karlin and Brendel, 1988; Chillon and Kremer, 2001).

For CAV to be an effective gene delivery tool, it should provide lasting, stable gene transduction. Indeed, to determine its viability for long-term experiments, Soudais et al. (2004) injected a CAV vector carrying green fluorescent protein (GFP) gene into multiple sites of the striatum in rats and indeed observed a high level of green fluorescent protein expression lasting for more than a year. In addition to rodents, CAV transduction led to a stable long-term transgene expression in a human stem-cell-derived 3D neural *in vitro* model (Simão et al., 2016), demonstrating its promise for functional experiments. Moreover, an ideal gene delivery vector should also avoid host immune responses and cytotoxicity. Indeed, CAV viruses are not human pathogens and do not induce significant cellular infiltration in rat brains (Soudais et al., 2004), nor in rhesus monkeys, unless abnormally high titers are used (Bohlen et al., 2019). Even a pre-existing immunity against human adenoviruses does not significantly affect CAV transduction (Klonjkowski et al., 1997; Kremer et al., 2000; Ibanes and Kremer, 2013). To further minimize the possible disruptions to normal neural processes caused by viral infection, CAV viral vectors were further engineered. For instance, the early region 1 (E1) gene, which is important for DNA replication, was deleted from CAV genome (Klonjkowski et al., 1997). The E1-deleted CAV is replication incompetent (Chartier et al., 1996; Fernandes et al., 2013) and thus causes negligible immune response in humans, non-human primates, and rodents at experimentally relevant titers (Kremer et al., 2000; Perreau and Kremer, 2005; Bohlen et al., 2019; Lau et al., 2019). Consequently, a large number of neurons can be transduced by E1-deleted CAV without being recognized and eliminated by immune cells (Kremer et al., 2000; Soudais et al., 2004; Perreau and Kremer, 2005; Lau et al., 2019).

Another line of evidence supporting the low cytotoxicity of CAV came from studies of neuronal morphology. CAV infection did not change the shape of the somata, the axonal arborization, the number of synaptic buttons, nor did it alter the ultrastructures of transduced neurons (Simão et al., 2016; Li et al., 2018). Interestingly, even with high multiplicity of infection (∼1,000 viral genomes/cell – which is 10–50× higher than normal; Hemmi et al., 1998), CAV did not disrupt the neuronal development of cultured cells, in contrast to both AAV and lentivirus (Piersanti et al., 2013). The long-lasting gene expression and negligible impact on the physiology of neurons make CAV a competent vector when expressing effectors for functional analyses. Indeed, CAV-Cre mediated the expression of effectors for chemogenetics (Augur et al., 2016; Roth, 2016; Alcaraz et al., 2018; Ramanathan et al., 2018), genetic ablation (Liu et al., 2016), and calcium imaging (Otis et al., 2017, 2019) for an intermediate time window, ranging from 2 weeks to a couple of months. Beyond 2 months, CAV can steadily express effectors for longer term functional analysis. For examples, CAV-mediated transduction of ChR2 in LC neurons remained stable for 6 months and was used to manipulate the sleep–wake transition of mice (Li et al., 2016). These experiments demonstrated the applicability of CAV for studying the physiology of neural circuits, with minimal effects on cellular health and circuit integrity.

## Applications

Canine adenovirus-2 is a powerful tool for mapping the input and output innervations of various types of projection neurons, and for recording or manipulating their activity. The simplest application of CAV involves transducing presynaptic and local neurons with either a fluorophore or an effector (Figure 1B). For instance, Li et al. (2016) revealed the existence of two separate neuronal populations in the rodent LC by injecting CAV carrying either red or green fluorophore in one of two known postsynaptic targets of LC neurons. Similarly, this straightforward experimental design was also used in non-human primates to express fluorescent proteins in neural pathways of interest, including motor neurons that innervate craniofacial muscles and midbrain neurons that project to the Cb (Bohlen et al., 2019; Dopeso-Reyes et al., 2019). This simple experimental design works well to tag projection neurons innervating a target of interest. However, on its own, CAV cannot be used to selectively target a single pathway, nor to report the anatomical origin of observed fluorescent axonal fibers. This limitation arises from the ambiguity that CAV can retrogradely transduce projection neurons non-specifically in any presynaptic region, as well as neurons near the injection site through their dendrites and somata, which express low but significant levels of CAR (Bru et al., 2010; Zussy et al., 2016; Dopeso-Reyes et al., 2019) and through their local axons (Figure 1B). To remove this ambiguity, a variety of conditional gene expression paradigms have been developed, taking advantage of DNA recombinases (Figures 1C–F; Nagy, 2000). For instance, CAV viruses carrying the Cre recombinase gene can be injected into a target of interest, in addition to a second injection of AAV viruses carrying a target gene flanked by loxP sequences, injected into a potential presynaptic site (Figure 1C). Cre recognizes loxP and conditionally turns on (or off) the loxP-flanked target gene (Nagy, 2000). By combining CAV’s retrograde capability with Cre-loxP conditional expression, this intersectional method (Figure 1A) labels specific neuronal projections (Senn et al., 2014; Penzo et al., 2015; Augur et al., 2016; Liu et al., 2016; de Git et al., 2018). This design was used to identify a corticofugal pathway from the VC to the brainstem and to ablate it exclusively (Liu et al., 2016), demonstrating the potential of CAV in projection pathway specific circuit analysis (Junyent and Kremer, 2015). The design in Figure 1C was ingeniously modified to express genes in an even more selective way (Figure 1D). In this new method, a second conditional expression system Flp–Frt (Flp, flippase recombinase; Frt, Flp recombinase target sequences) (Rodríguez et al., 2000) is added so that a Cre transgenic mouse line is used to restrict the expression of target genes in molecularly defined cell types, while Flp carried by CAV selects projection pathways. Therefore, both projection specificity and cell-type specificity are accomplished simultaneously with this clever design. This intersectional method was used in a few studies to lay out the projections of specific types of neurons (Schwarz et al., 2015; Chung et al., 2017; Kakava-Georgiadou et al., 2019). For example, Chung et al. (2017) examined the output pattern of GABAergic neurons in the preoptic area projecting to the TMN pathway.

The above intersectional strategy is not limited to the applications of mapping direct monosynaptic connections between two brain regions (a→b) (Figures 1C,D). Instead, it can also be applied to trace more complex neural circuits involving disynaptic connections (a→b→c), with the help of EnvA-pseudotyped, glycoprotein (G)-deleted rabies viruses (RVdG) (Figures 1E,F). Wild-type rabies viruses are capable of transsynaptic transport, which allows them to move from postsynaptic neurons, via synapses, to presynaptic neurons (Wall et al., 2010). The engineered RVdG, however, loses this capability unless the target postsynaptic neurons (the so-called starter cells) express both avian sarcoma leukosis virus receptor (TVA) and G (Wall et al., 2010). TVA receptor is required for EnvA-pseudotyped rabies to enter starter cells, and G protein is necessary for rabies’s transsynaptic capability (Wall et al., 2010). The expression of those two proteins in starter cells complements RVdG and allows it to infect the starter cell’s presynaptic neurons (Wall et al., 2010). Making use of this elegant design, the disynaptic tracing outlined in Figures 1E,F occurs in two steps: (1) the intersectional method detailed previously, which uses CAV-Cre to selectively express TVA and G protein in starter cells, defining the output projections (b→d); (2) the retrograde transsynaptic transport of RVdG determines the input innervation of starter cells (a→b). These disynaptic tracing methods have been used to determine the pattern of inputs received by individual noradrenaline populations in LC defined by their output target and cell type (Schwarz et al., 2015). This study benefited greatly from CAV’s large size and low diffusion rate, which confines CAV-Cre within the size of small brain regions of interest (Schwarz et al., 2015). In sum, CAV is most widely used to carry recombinases or recombinase-dependent constructs (Table 1), making it a powerful retrograde vector to investigate both monosynaptic and disynaptic connections within a neural circuit.

Even though most of our examples of CAV and intersectional method applications concerned circuit anatomy, the same intersectional approaches can be easily tweaked for functional analyses or molecular profiling. For instance, a variety of effector molecules have been conditionally expressed for either chemogenetics (Boender et al., 2014; Augur et al., 2016; Alcaraz et al., 2018; Fernandez et al., 2018; Ramanathan et al., 2018; Kakava-Georgiadou et al., 2019), optogenetic (Eliava et al., 2016; Li et al., 2016), projection-specific genetic ablations (Wu et al., 2012; Liu et al., 2016), optical calcium imaging (Otis et al., 2017, 2019), and molecular profiling (Ekstrand et al., 2014). Furthermore, the ability of CAV to seamlessly deliver and selectively express effectors can be paired with classical techniques, such as slice or *in vivo* electrophysiology (Eliava et al., 2016; Li et al., 2016), and a variety of behavior paradigms (Liu et al., 2016; Kakava-Georgiadou et al., 2019). Pairing these techniques with CAV and intersectional methods enables a wide range of combinations, allowing substantial versatility and creativity in experimental designs. It should be noted that, regardless of the vector used, long-term overexpression of Cre recombinase can be toxic (Silver and Livingston, 2001; Braz et al., 2002; Whitsett and Perl, 2006; Gong et al., 2007; Harno et al., 2013; Janbandhu et al., 2014; Lam et al., 2019), and therefore, precautions are needed when Cre is used in functional assays.

## CAV Limitations and Future Directions

In spite of the great success of CAV in the above studies, one should be cautious of the limitations of this tool and aware of the caveats when interpreting experimental results. For instance, there is evidence that CAV transduction biases some types of neurons or pathways (Table 2), likely due to varying CAR expression level in different types of neurons (Kremer, 2004; Grove and Marsh, 2011). For example, when examining the projection from basolateral Amy (BLA) to mPFC, Senn et al. (2014) found that CAV and herpes simplex virus 1 retrogradely labeled largely non-overlapping subpopulations of BLA neurons when injected into the mPFC area, indicating that CAV transduced only a part of this projection population. In addition, the strong tropisms of CAV for particular cell types over others was also observed in corticocortical projections. In particular, CAV injected in the ACC of mice preferentially transduced neurons in layer 5 of the primary VC; in contrast, other retrograde viruses, such as engineered rabies and rAAV2-retro, injected in the same cortical area transduced neurons across multiple layers of the primary VC (Chatterjee et al., 2018). Therefore, when using CAV to map unknown connectivity, one must be cautious when drawing conclusions about negative results, as a lack of evidence for connectivity could be alternatively explained by CAV tropism.

**TABLE 2:** Summary of cell type or projection-specific CAV tropism.

System	Circuit	Comparison	References
Glutamatergic	BLA → mPFC	CAV: bias medial BLA cells HSV: bias lateral BLA cells Retro-beads: no bias	Senn et al., 2014
	(contra. ACC, ORBvl, VISal, RSP) → ACC	CAV: bias L5 cells Retro-AAV: bias L2/3 cells Rabies: both L5 and L2/3 cells	Chatterjee et al., 2018
	Cortices → BPN	CAV: low efficiency rAAV2-retro: high efficiency Fluro-Gold: high efficiency	Tervo et al., 2016
	OFC → VS	CAV: low efficiency CAV + AAV-CAR: high efficiency	Li et al., 2018
	BLA → mPFC	CAV: low efficiency CAV + AAV-CAR: high efficiency	Kakava-Georgiadou et al., 2019
Dopaminergic	(VTA, DNC) → DLS	CAV + AAV-CAR: high efficiency rAAV2-retro: low efficiency	
	VTA → NAc	CAV = CAV + AAV-CAR: high efficiency rAAV2-retro: low efficiency	

A second limitation of CAV is its efficacy of retrograde transduction. While sufficient to drive gene expression for both anatomical and functional experiments (Table 1), CAV viruses are not quite comparable in infectivity and retrograde transport to other retrograde viruses such as rAAV2-retro and rabies virus (Aschauer et al., 2013; Tervo et al., 2016; Chatterjee et al., 2018; Table 2). For example, in the corticopontine circuit, rAAV2-retro viruses injected into the basal pontine nuclei transduced 22 times more layer 5 projection neurons in the cortex than the CAV viruses (Tervo et al., 2016). As such, a more efficient CAV virus with little tropism would be a great improvement, since it could facilitate the detection of sparse projections and increase the strength of functional manipulations, facilitating the detection of subtle physiological or behavioral effects.

To improve the efficiency of CAV or even overcome its tropism against some neuronal types, an elegant receptor complementation strategy was recently developed, focusing on CAR, a key receptor molecule for the retrograde capability of the CAV (Li et al., 2018). In this method, the CAR receptor was virally expressed in candidate projection neurons, which in turn facilitated the retrograde transduction of those targeted neurons by CAV carrying Cre recombinase (Li et al., 2018). This strategy increased the efficacy of CAV retrograde transport in the BLA-to-mPFC pathway, where CAV has limited infectivity in control animals (Li et al., 2018). This strategy is also potentially useful to circumvent the tropism of other retrograde viruses, providing CAR/CAV combination a significant advantage over rAAV2-retro and rabies (Li et al., 2018). For example, rAAV2-retro viruses showed marginal retrograde transduction of dopaminergic neurons in the VTA and SNc (VTA/SNc) that project to dorsolateral striatum (DLS) (Tervo et al., 2016). By virally expressing the CAR receptor in VTA/SNc, the number of dopaminergic neurons that were retrogradely infected by the CAV injected into the DLS was approximately nine times larger than when rAAV2-retro was used. Nevertheless, the applicability and limitations of this receptor complementation strategy in other cell types and pathways need to be explored and validated (Kakava-Georgiadou et al., 2019).

## Conclusion

In summary, the unique characteristics of CAV make it an intriguing choice for neuroscientists. CAV vectors complement the toolbox of retrograde viruses, which can be used to reveal the connectivity and physiology of neural circuits. In particular, due to low toxicity, minimal immunogenicity, and stable gene expression, CAV is ideal for long-term functional analyses of brain circuits. Moreover, the capability of combining CAV with conditional expression and transsynaptic tracing makes it a promising tool to study circuits in cell- and/or projection-type specific manners. Finally, the success of the CAR receptor complementation strategy provides a method to circumvent the limitations of CAV, opening a new era for circuit analysis. Needless to say, CAV will continue to facilitate the long-standing quest to ultimately understand the biological substrates and logics of brain functions.

## Author Contributions

Both authors contributed to the writing and figure design.

## Conflict of Interest

The authors declare that the research was conducted in the absence of any commercial or financial relationships that could be construed as a potential conflict of interest.

## Abbreviations

ACC: anterior cingulate cortex
ACtx: auditory cortex
Amy: amygdala
Ath: auditory thalamus
BLA: basolateral amygdala
BPN: basal pontine nucleus
Cb: cerebellum
CeA: central amygdala
CLA: claustrum
CoA: cortical amygdaloid nucleus
Contra.: contralateral
DBB: diagonal band of brocca
DCN: deep cerebellar nuclei
DG: dentate gyrus
DMH: dorsomedial hypothalamus
DRN: dorsal raphe nucleus
EC: entorhinal cortex
GC: gustatory cortex
HPC: hippocampus
IC: inferior colliculus
IO: inferior olive
LC: locus coeruleus
LH: lateral hypothalamus
LHb: lateral habenula
LS: lateral septum
LTDg: laterodorsal tegmentum
M1: primary motor cortex
MC: motor cortex
MdD: reticular formation
MDT: medial dorsal thalamus
Me: medulla
mPFC: medial prefrontal cortex
mPOA: medial preoptic area
NAc: nucleus accumbens
NG: nodose ganglia
NOT/DTN: complex of nucleus of optic tract and dorsal terminal nucleus
NRTP: nucleus reticularis tegmenti pontis
NTS: nucleus tractus solitaries
OB: olfactory bulb
OFC: orbitofrontal cortex
ORBvl: ventrolateral orbital cortex
PAG: periaqueductal gray
PBNl: lateral parabrachial nucleus
PCRt: parvocellular reticular formation
PFC: prefrontal cortex
PLC: prelimbic cortex
POm: posterior medial thalamic nucleus
preBötC: preBötzinger complex
PVN: hypothalamic paraventricular
PVT: paraventricular thalamus
RE: thalamic nucleus reuniens
RSP: retrosplenial cortex
SC: superior colliculus
SNc: substantia nigra pars compacta
SON: supraoptic nuclei
STN: subthalamic nucleus
TMN: tuberomammillary nucleus
VC: visual cortex
vMT: ventral midline thalamus
VTA: ventral tegmental area.
